# Intelligent Monitoring and Visualization System for High Building Nighttime Utilization Based on Image Processing

**DOI:** 10.3390/s24216793

**Published:** 2024-10-22

**Authors:** Yuanrong He, Xianhui Yu, Qihao Liang, Peng Yu, Zhiying Xie, Le Xia, Minghang Qin

**Affiliations:** 1 Big Data Institute of Digital Natural Disaster Monitoring in Fujian, Xiamen University of Technology, Xiamen 361024, China; 2012112001@xmut.edu.cn (Y.H.); 2222031169@stu.xmut.edu.cn (X.Y.); 2222041004@stu.xmut.edu.cn (Q.L.); 2020000042@xmut.edu.cn (Z.X.); 2322091004@stu.xmut.edu.cn (M.Q.); 2 Hunan Key Laboratory of Remote Sensing Monitoring of Ecological Environment in Dongting Lake Area, Changsha 410004, China; jacky_xiale@163.com

**Keywords:** real-scene 3D model, geographic visualization, nighttime detection, image processing, smart campus

## Abstract

The rise of complex high-rise buildings has made building management increasingly challenging, especially the nighttime supervision of university laboratories. Idle occupation increases the risk of accidents and undermines campus sustainability. Effective occupancy detection is essential for optimizing campus building safety and energy efficiency. Environmental sensors for occupancy detection offer limited coverage and are costly, making them unsuitable for campuses. Surveillance cameras, as part of campus infrastructure, provide wide coverage. On this basis, we designed a detection algorithm that uses light brightness to assess nighttime building use. Experimental results showed that the algorithm achieves an average accuracy of 98.67%, enabling large-scale nighttime occupancy detection without the need for installing additional sensors, significantly improving the efficiency of campus building management. In addition, to address the limitations of indoor space representation in geographic information system (GIS) management models, this paper developed a comprehensive 3D GIS model based on a “building–floor–room” hierarchical structure, utilizing oblique photogrammetry and laser scanning technology. This study combined the detection results with real-world 3D data for visualization, providing a new perspective for the 3D spatiotemporal refinement of complex high-rise buildings, and providing a reference framework for the detection and analysis of other types of building environments.

## 1. Introduction

Over the past 20 years, the number of students enrolled in Chinese higher education has reached 47.6319 million [1], leading to a conflict between universities’ limited land resources and the growing demand for teaching and research facilities [2]. In response, universities are increasingly constructing vertical high-rise buildings with complex internal layouts to meet these needs [3,4]. However, this intricate mixed-use design significantly heightens spatial management challenges [5]. GIS and building information modeling (BIM), as three-dimensional (3D) data carriers, play a crucial role in smart city management, facilitating spatiotemporal data acquisition, storage, spatial analysis, and visualization [6]. They have proven to be highly effective in urban planning and design [7], facility management [8], disaster emergency management [9], and public service optimization [10]. Moreover, advancements in deep learning and machine learning [11] have enhanced the application of image processing in smart buildings, significantly improving the perception analysis efficiency [12]. The integration of image processing technology with GIS and BIM models provides robust support for sustainable development and intelligent building management.

Building occupancy detection is crucial for enhancing safety, energy efficiency, and environmental comfort [13]. However, poor management has made university buildings high-risk areas for accidents, especially during the nighttime [14]. Nighttime management typically relies on manual inspections, which hamper effective management [15]. Therefore, employing sensing systems to detect nighttime occupancy in university buildings is essential for ensuring campus safety and improving management efficiency.

Sensing systems for intelligent occupancy detection can be categorized into data-driven methods and image-based methods [16,17]. Most research has been focused on commercial or residential buildings, leaving a gap in studies related to university architectures [18]. Data-driven sensing systems, such as environmental and motion sensors, can monitor occupancy and occupant behavior effectively [19]. For instance, radio frequency identification (RFID) [20], passive infrared sensors (PIRs) [21], and infrared sensors [22] can track building usage. However, these systems require installation and maintenance and have limited coverage, making them less ideal for large campus environments. In contrast, wireless networks (Wi-Fi) serve as an integral part of campus infrastructure, offering wide coverage without the need for extra installation. Nevertheless, practical applications of Wi-Fi can be hindered by network disconnections, device shutdowns, and signal obstructions, which limit the effectiveness of nighttime detection. Alternatively, image-based sensing systems, such as continuous monitoring equipment of campus infrastructure combined with image processing technology, offer broader applications. Real-time occupancy detection can be achieved by analyzing indoor monitoring images [23,24]. However, for complex high-rise buildings, the real-time analysis of a large volume of monitoring images requires substantial computing resources, particularly for complex visual tasks [25]. This limitation impacts the generalizability and cost-effectiveness of this approach [26].

Currently, campus management visualization primarily relies on two-dimensional data such as monitoring systems and tables [27,28]. These include examples like thermal and solar energy assessments [29], building occupancy detection [30], energy consumption analysis [31], vulnerability assessments [32], and laboratory safety management evaluations [33]. However, due to the high density and mobility of personnel on campus, spatiotemporal visualization is crucial for effective management decision-making. Francisco et al. [34] proposed specific and accurate time-segmented building efficiency measurement standards, but they overlooked the analysis of occupied spaces. Piselli et al. [35] developed a data-driven occupancy model, but it was limited to flat buildings with simple geometric structures. Zhao et al. [36] utilized geographic models for macroscopic building occupancy visualization, but they lacked microscopic-scale visualization models. The 3D GIS provides a foundational data framework for campus management visualization [37,38,39]. However, GIS primarily focuses on surface models and lacks detailed representations of building interiors [40]. As faculty and students spend 80% of their time indoors, indoor space data have become vital for spatiotemporal analysis, making precise indoor space management increasingly urgent [41]. BIM offers flexible indoor spatial information [42], which is beneficial for managing complex and densely populated campus buildings.

This study presents an innovative and cost-effective method for nighttime occupancy detection, integrating outdoor surveillance video image processing, to address the prohibitive costs associated with existing complex occupancy detection systems in high-rise buildings. The approach begins with the evaluation of window brightness extraction from outdoor monitoring images. To enhance the accuracy, this method incorporates indoor people detection to effectively identify unoccupied rooms. To address the challenges of campus visualization and the lack of indoor spatial data in current GIS frameworks, we propose a comprehensive approach for constructing outdoor 3D GIS models and multi-scale hierarchical representations of indoor spaces. Utilizing advanced laser scanning and oblique photography technologies, we develop a comprehensive 3D model that integrates both indoor and outdoor environments, creating a “room-level” database optimized for detection applications and informed decision-making in educational management.

The primary contribution of this research is a dual-faceted method that leverages both indoor and outdoor surveillance videos for detecting nighttime occupancy in university buildings, significantly reducing computational demands for occupancy assessment in high-rise structures. Furthermore, a 3D multi-scale building model is presented to elucidate spatial usage at the “room level”, enhancing its application for campus management and planning.

The remaining organizational parts of the paper are as follows: Section 2 introduces the research methods, including the acquisition and processing of sensor data, the construction of real-time 3D models, nighttime use detection algorithms, and the visualization process of detection results. In the Section 3, the system displays the occupancy patterns over different time periods and the internal situation of the laboratory, facilitating the management, analysis, and application of geographic data. The Section 4 discusses the results, and the Section 5 draws some conclusions and proposes future research advice.

## 2. Materials and Methods

**Figure 1:**
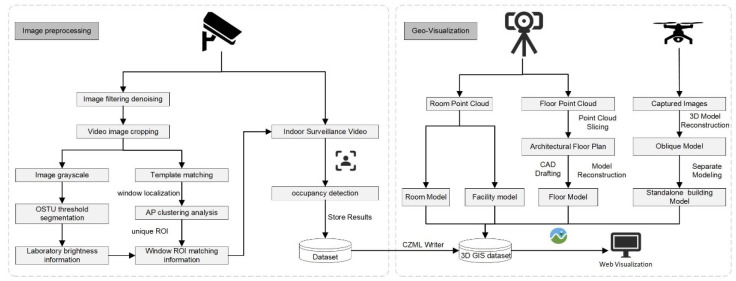
Methodology framework.

This study consists of two parts: image processing and geographic visualization (Figure 1). Firstly, oblique photography and LiDAR scanning technologies are used to collect geographic data and generate 3D geographic models. Next, indoor and outdoor monitoring images are obtained through surveillance, and image processing techniques are used for template matching, clustering analysis, threshold segmentation, and object detection to extract building usage information. Finally, we visualize the application on web clients.

### 2.1. Internet of Things Perception Data

**Figure 2:**
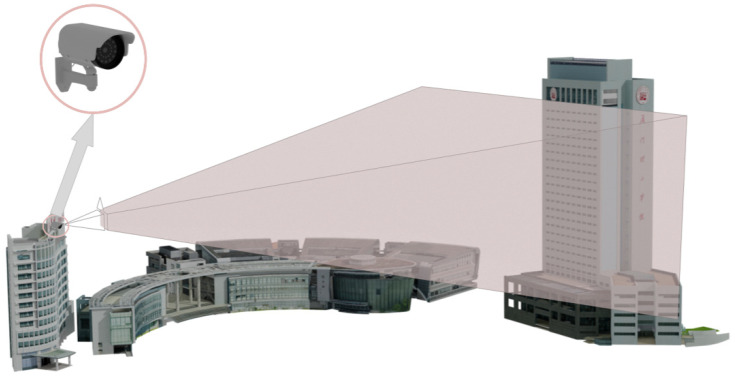
Diagram illustrating video data acquisition.

The research focuses on the comprehensive building at Xiamen University of Technology, which has 25 floors, a height of 120 m, and a total area of 48,577 m^2^. It primarily serves as a hub for research, teaching, office, and training activities. This study chose the DAHVISION network surveillance camera, and the sensor parameters are detailed in Table 1. The network camera is connected to the streaming host through the network and transmits data using the RTSP protocol for subsequent image processing and analysis. The video surveillance system is installed on the roof outside the building, as shown in Figure 2.

### 2.2. Acquisition and Processing of Three-Dimensional (3D) Geographic Data

Campus 3D models are the foundation of geographic visualization. However, due to the involvement of multiple aspects such as geometry, semantics, appearance, and attributes, creating a universal 3D building model that accommodates various scales, like a 2D topographic map, is difficult. This article focuses on the specific needs of high-rise building management and nighttime occupancy detection by establishing a multi-scale realistic 3D model with the “Building–Floor–Room” organization.

**Figure 3:**
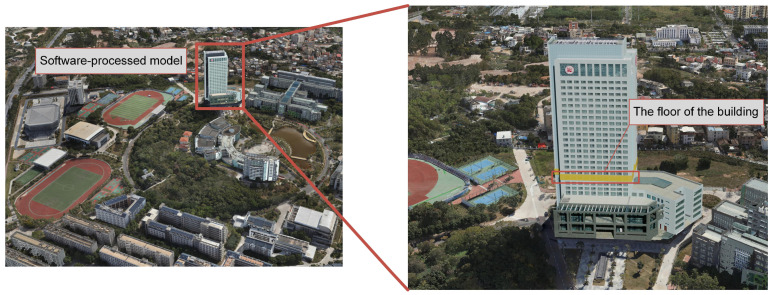
Building a model using oblique photography.

Firstly, the study utilizes unmanned aerial vehicle (UAV) oblique photography technology to quickly construct a realistic 3D oblique model surrounding the target building using Context-Capture software (V4.2.23) and DP Modeler software (V2.3) to remove the scene and building defects and to construct the model on a “block” basis. The 3D model of the target building is illustrated in Figure 3.

**Figure 4:**
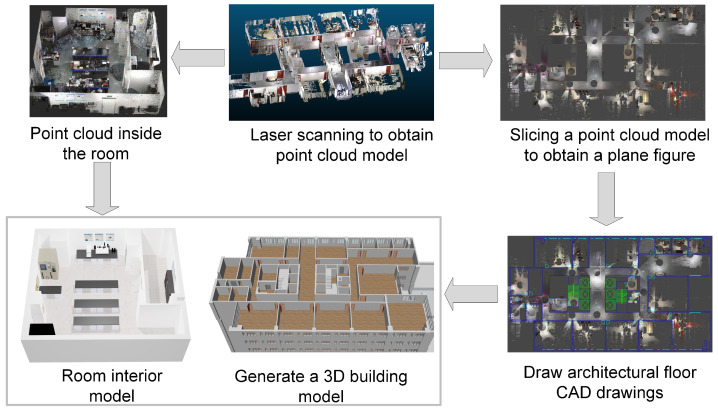
Flowchart for constructing floor models and indoor models using laser scanning technology.

To build the floor models, a laser scanner is used to capture indoor point clouds of the target building. The output point cloud undergoes processing, including registration and noise reduction. The point cloud is then sliced to create indoor plane models, followed by the creation of CAD drawings based on these slices. Using the Blender-BIM plugin, we incorporate component information such as walls, doors, and windows into the indoor plane models, generating the 3D model of each floor in “layers”. The Blender software V3.6.4 enables point cloud rendering, allowing us to construct indoor and equipment space models of the laboratory, and adding equipment attribute information to 3D output models of different functional rooms in “household” units. The process of building floor models and indoor models is depicted in Figure 4.

Finally, spatial vector data for the building’s external windows are required, and each window in the monitoring screen corresponds to laboratory vector data. The window vector parameters are filled with corresponding attributes, such as window code, laboratory name, and laboratory code, before being output in GeoJSON format. This enables the computer to interpret the semantic data of the 3D model and achieve the mapping from reality to virtual representations.

### 2.3. Night Detection Algorithm Based on Image Processing

**Figure 5:**
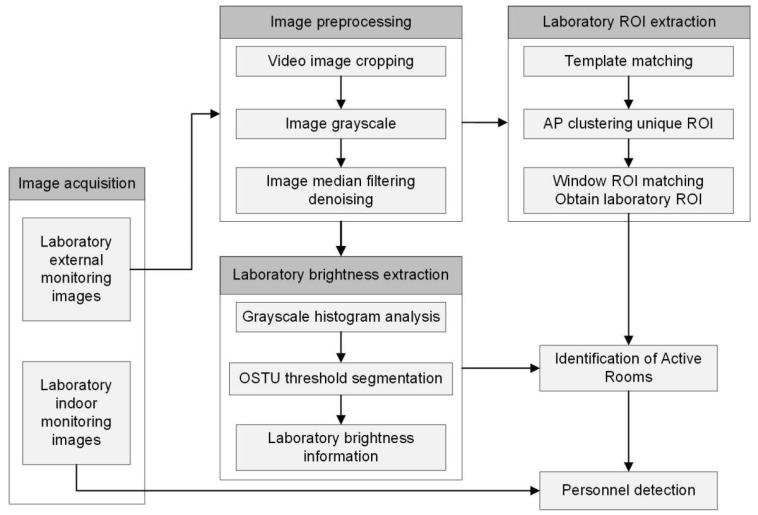
Workflow for detecting laboratory’s nighttime usage status.

By extracting lighting information from nighttime monitoring, we can determine whether the laboratory is in use. Given the fixed shooting area of the surveillance camera and the clarity of the daytime images, it is possible to capture daytime images to identify the positions of laboratory windows and extract laboratory brightness information from the nighttime monitoring images. Combining these two datasets enables real-time monitoring of the laboratory’s nighttime usage status. The specific process is depicted in Figure 5.

First, the outdoor monitoring images undergo preprocessing, including study area cropping, noise reduction, and grayscale processing. Next, brightness extraction is performed, and the OSTU algorithm is used for threshold segmentation, following grayscale histogram analysis. In addition, template matching is performed using well-lit daytime images to identify the positions of all windows. Then, the threshold segmentation results are combined with template matching to locate the regions of interest (ROIs) in the laboratory. Finally, indoor monitoring data are obtained for human occupancy detection, allowing us to determine laboratory usage based on the results of two tests.

#### 2.3.1. Image Preprocessing

To address the high resolution and complexity of surveillance video images, image preprocessing is performed to eliminate irrelevant information, such as non-laboratory areas and external building lighting sources. Image preprocessing involves cropping, converting to grayscale, and applying median filtering for denoising, ultimately enhancing the speed and accuracy of detection algorithms.

**Figure 6:**
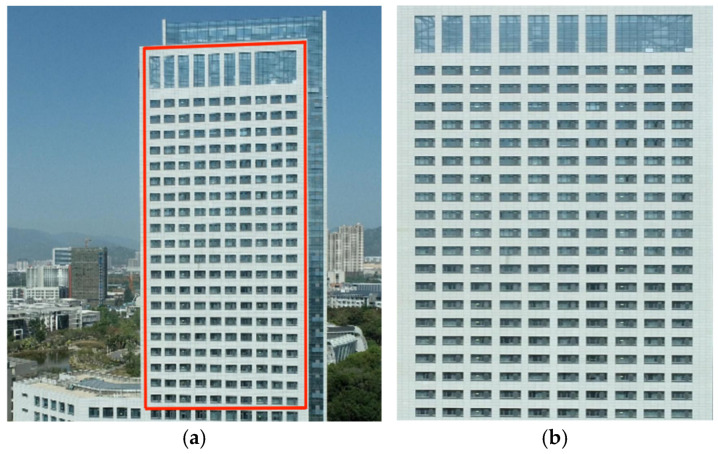
Monitoring image (**a**) before and (**b**) after cropping.

To address the irrelevant information in the captured video image, the first step is to crop the region image. The image of the research area is cropped using perspective transformation. By manually selecting four key points of the laboratory area in the video surveillance screen, we calculate the perspective transformation matrix and reproject the sub-image onto a matrix image screen to crop the video image of the laboratory area. The perspective transformation allows us to process the image that only includes the research area, which can reduce the computational complexity. Figure 6a shows the original monitoring image, with the red box indicating the perspective transformation area. Figure 6b shows the cropped monitoring image.

After cropping, the output BMP format image is an RGB three-channel image with a larger data volume. To reduce memory resource consumption and improve processing efficiency, grayscale processing is applied to the image. The maximum method has low image contrast, while the average method results in blurred edges and loss of detail. Therefore, this study employs the weighted average method for image grayscale processing [43], as depicted in Equation (1).

Gray(x, y) = 0.3(x, y, 1) + 0.59(x, y, 2) + 0.11(x, y, 3)(1)

where Gray(x, y) represents the grayscale value at the point (x, y). The weights are designed based on the human body’s sensitivity to RGB, with the values of 0.3 for R, 0.59 for G, and 0.11 for B. This method keeps higher overall brightness and more details, and is thus suitable for the proposed detection algorithm.

After grayscale processing, image filtering and noise reduction are required to reduce the noise caused by electronic devices and networks. The average filtering and Gaussian filtering can cause blurring of window edges and loss of details. Our study adopts median filtering (Equation (2)) for noise reduction, which is a kind of nonlinear filtering. It replaces the grayscale values of (x, y) pixels with the median grayscale values of all pixels in the region M centered on it [44]. The size of the area is m × n, which can be manually adjusted, but the area M should not be too large.

F(x, y) = Median[g(i, j)],(i, j)∈M_xy_ (2)

(i, j) is the pixel position relative to the center. Taking the median of the pixel values, we can effectively eliminate extreme salt and pepper noises. This filter can effectively remove noise while preserving window details in the image, which is beneficial for subsequent template matching to extract laboratory ROI localization. Therefore, median filtering is chosen for image denoising.

#### 2.3.2. ROI Extraction of Room Windows

The accurate positioning of laboratory windows is crucial for precise detection and visualization. To efficiently extract window positions from a large dataset, this study employs template-matching algorithms, using the normalized cross-correlation (NCC) algorithm for similarity measurement.

**Figure 7:**
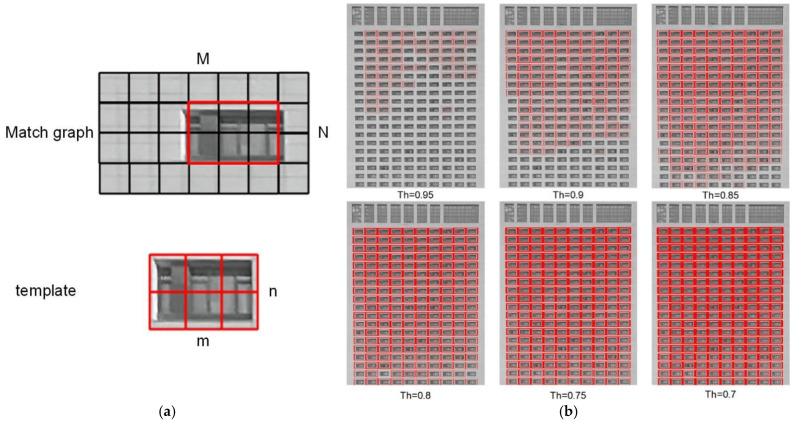
Template matching. (**a**) The obtained window image used as a matching template. (**b**) Result of window matching at different thresholds.

We use daytime surveillance videos as matching images. After preprocessing, a clear window image is randomly selected as a matching template, as shown in Figure 7. Multiple thresholds are manually set to evaluate the matching performance of the NCC algorithm and to determine the optimal threshold.

**Figure 8:**
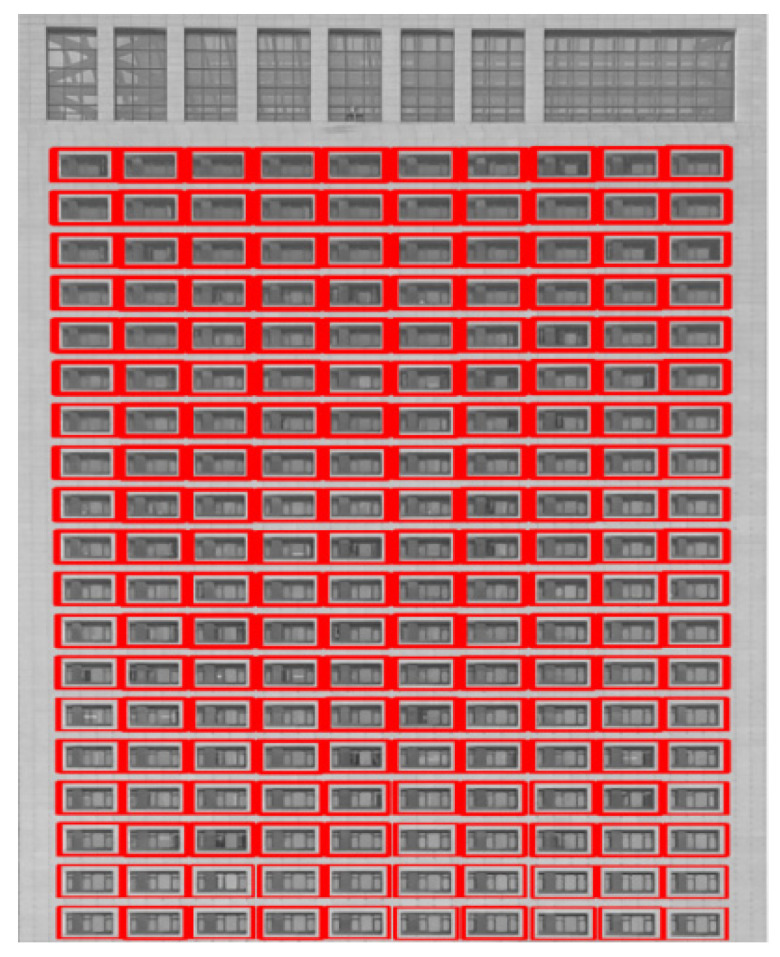
Window matching results at a threshold of 0.75.

The NCC matching algorithm is affected by changes in external lighting and has strong resistance to white noise. As shown in Table 2, a threshold of 0.75 successfully matched all windows with fewer repeated matches compared to a 0.70 threshold. Therefore, this study uses this value to obtain window positioning coordinates. Figure 8 shows the template-matching results at a threshold of 0.75.

**Figure 9:**
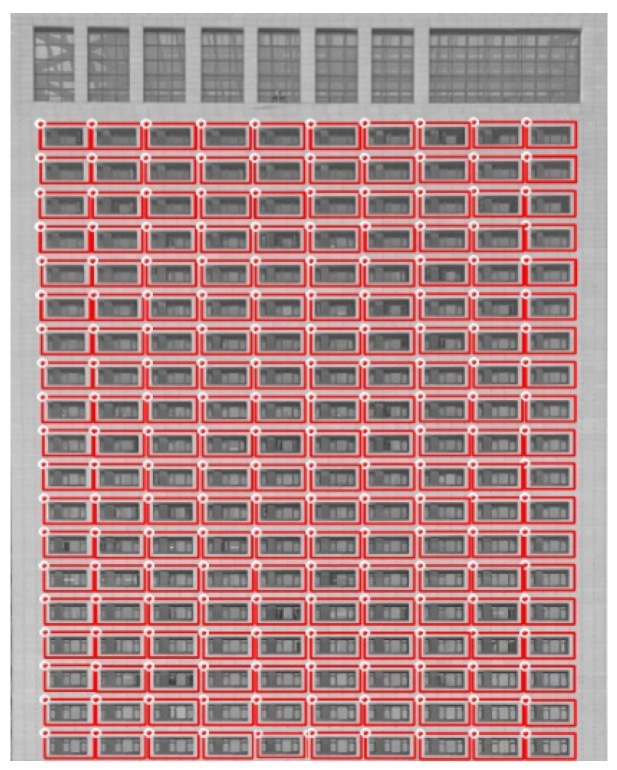
Clustering analysis results.

The table above shows a duplicate matching issue within the NCC template matching, as each laboratory window should only have a unique and accurate position. The nearest-neighbor propagation clustering method does not require specification of the number of clusters in advance and can effectively handle data clustering problems with a total number of elements greater than 900. Therefore, it is used to obtain the unique coordinates of each window. The key parameters (damping coefficient λ, maximum iterations, and convergence iterations) are set to 0.5, 300, and 5, respectively. Clustering results are shown in Figure 9.

The image reveals a substantial repetition of window textures. Through clustering analysis, 190 coordinate matrices were identified as the unique ROIs for laboratory recognition. Each ROI is then matched with specific laboratory information for detection algorithms. For instance, if the window’s coordinate array is “[[21,290], [45,310]]”, it is mapped in the system to the laboratory window ID “SYLSYSCH0051” and the laboratory ID “SYLSYS2011”. These data are recorded in the system database as a distinct dataset.

#### 2.3.3. Nighttime Brightness Extraction

**Figure 10:**
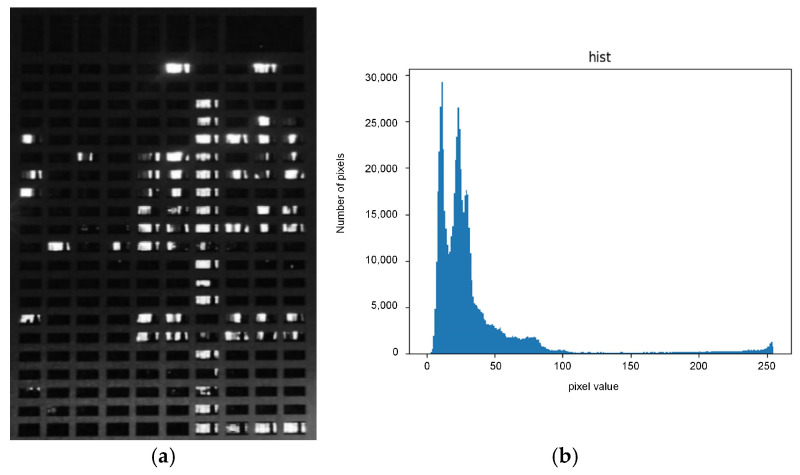
(**a**) Night surveillance video image and (**b**) image grayscale histogram. The horizontal axis represents the grayscale level of the image pixels, with a distribution interval of [0, 255], and the vertical axis represents the total number of pixels at that grayscale level.

After image preprocessing and window position identification, the image is further grayed out for subsequent nighttime brightness extraction and usage status judgment. In the night images after grayscale processing, whiter areas indicate higher grayscale values and brightness. The grayscale histogram can be used to analyze the distribution of grayscale levels and plays a crucial role in determining the threshold for histogram segmentation. Figure 10a,b show the grayscale image and histogram of the nighttime surveillance video.

As Figure 10 shows, most pixel grayscale values are less than 50, indicating the image is generally dark. The pixel grayscale values of the laboratory in use are concentrated in the interval [20, 255].

**Figure 11:**
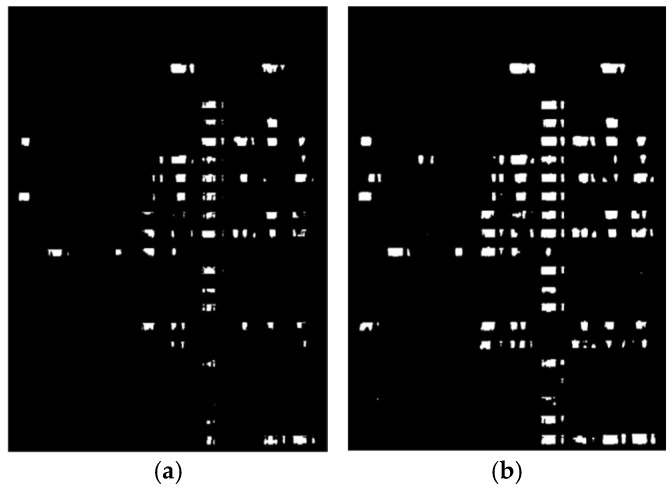
Comparison of brightness information extraction result. (**a**) shows the histogram threshold segmentation result with a threshold of 150, and (**b**) shows the OSTU threshold segmentation result with a threshold of 112.

The histogram threshold segmentation method obtains its segmentation threshold by analyzing the grayscale histogram of the image. According to Figure 10b, there is a significant difference in grayscale between the highlighted and low-brightness areas. Combined with Figure 10a, the highlighted regions in the histogram are the main focus of this study. In addition, Figure 10b shows that the peak of the low-brightness area appears before a grayscale value of 100, and the peak of the high-brightness area appears after 200. The valley between the peaks of the highlighted and non-highlighted regions is at a grayscale value of 150. Therefore, this study uses a threshold of 150 to perform histogram threshold segmentation on the image in the future. As Figure 11a shows, this is the result of the histogram thresholding method. OSTU threshold segmentation (OSTU) is an adaptive thresholding method that can automatically calculate the optimal segmentation threshold. We use the OSTU threshold segmentation algorithm for the image in Figure 11a and obtain a segmentation threshold of 112. The result is depicted in Figure 11b below.

Figure 11a shows that a threshold value of 150 results in a significant loss of brightness information. In contrast, Figure 11b, segmented using the OSTU algorithm, preserves all laboratory brightness information, yielding a more complete segmentation result. Therefore, this study uses te OSTU threshold segmentation method to extract laboratory brightness information.

#### 2.3.4. Detection Algorithm of Laboratory Night Use Based on Outdoor Monitoring

This study utilizes laboratory ROI localization and real-time nighttime brightness information from surveillance videos to detect laboratory nighttime usage status. The process begins with loading the obtained laboratory ROI location to determine the laboratory to which a lighting window belongs. The algorithm takes less than 1 s to detect an image, processing one image per second to maintain the timeliness required for real-time detection. By analyzing the average grayscale value within the laboratory ROI, we determine whether the laboratory will be used in the future. If the average grayscale exceeds 30, it indicates that one-eighth of the laboratory window area is bright, indicating that the laboratory is in use.

#### 2.3.5. People Detection Algorithm Based on Indoor Monitoring

For laboratories deemed to be in use, traditional manual monitoring and inspection can lead to human errors, especially during nighttime fatigue, and multiple tasks. To address this, this study proposes a deep learning YOLOV8 model for indoor people detection, complementing the outdoor nighttime lighting laboratory usage detection. This approach aims to improve the efficiency of safety management and reduce the risks caused by human errors.

**Figure 12:**
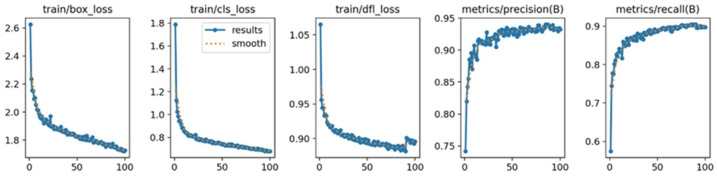
Details of model training: loss and evaluation metrics. The train/box_loss plot shows the changes in bounding box regression loss during training, the train/cls_loss plot illustrates the loss variation in the classification task, and the train/dfl_loss plot depicts changes in distribution focal loss. Additionally, the matrices/precision(B) plot represents the precision changes throughout training, while the matrices/recall(B) plot shows the recall rate variation.

YOLOv8 [45] is a widely used detection algorithm in the YOLO series, which can quickly complete detection, classification, and segmentation tasks [46]. This study collects laboratory video surveillance footage as a dataset, consisting of 2000 images—1600 for training and 400 for validation. We use transfer learning methods to train YOLOv8n, as detailed in Table 3. The training parameters included a learning rate of 0.01, 100 epochs, and a batch size of 2. Figure 12 illustrates the model training details. 

The effectiveness of the nighttime detection model is assessed by precision (P). The recall rate, indicating the model’s ability to identify personnel targets in the image, reflects the model’s accuracy. Detection speed mainly assesses the model’s capability to process images within a unit of time, effectively measuring its performance in practical applications. The equation for calculating precision is presented in Equation (3).

(3) $$\mathrm{precision}=\frac{\mathrm{TP}}{\mathrm{TP}+\mathrm{FP}}$$

where TP (true positives) refers to the number of people correctly detected by the algorithm when present in the image. Conversely, FP (false positives) denotes the number of people misidentified by the algorithm when not present in the image.

In the actual detection process, this study packages and deploys deep learning models on local servers. It uses the OpenCV library to read image streams from surveillance video sources, capturing images at a rate of one frame per second. These images are then used for model detection, with the results stored in a local database. For laboratories identified as in use, the system analyzes the monitoring videos and employs deep learning algorithms to detect personnel indoors.

After training, the internal monitoring videos can be read to determine the number of personnel in each image and judge the laboratory usage based on the personnel situation.

### 2.4. Visualization of Test Results

**Figure 13:**
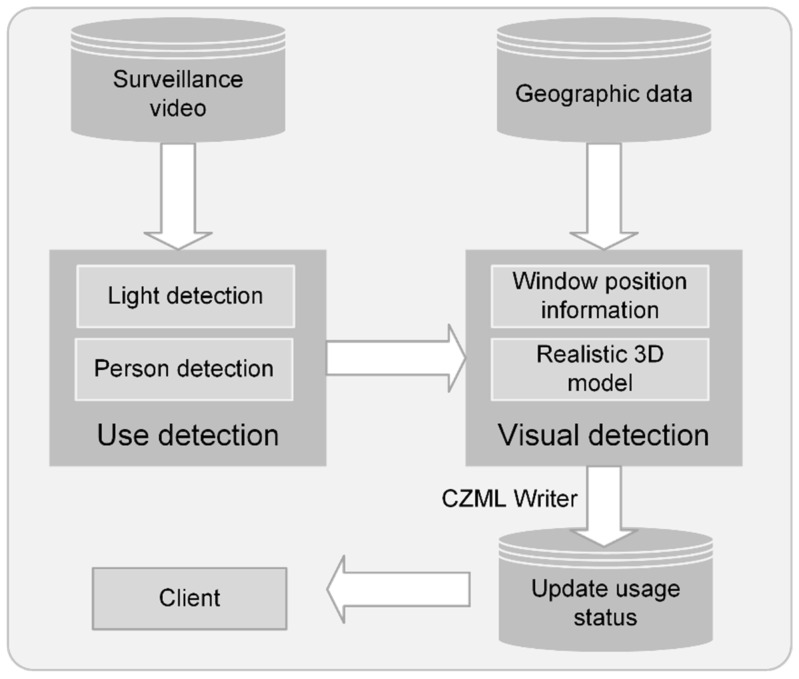
Geographic visualization workflow of the detection results.

Cesium is a JavaScript library for cross-platform, cross-browser 3D Earth and map visualization. The Cesium language (CZML) can describe dynamic scenes and transmit streaming data online. In this study, Cesium is chosen as the platform for loading real-life 3D models. Firstly, the OSGB and OBJ files of the 3D models are converted into 3DTiles format data using the software Cesiumlab and loaded to the same coordinate system. According to the detection results, we attach timestamps and color attributes to the window vector data, then update and dynamically render them to the 3D model based on the timestamp of the detection results. The workflow is depicted in Figure 13.

## 3. Results

### 3.1. Nighttime Detection of Laboratory Use Based on Outdoor Monitoring

**Figure 14:**
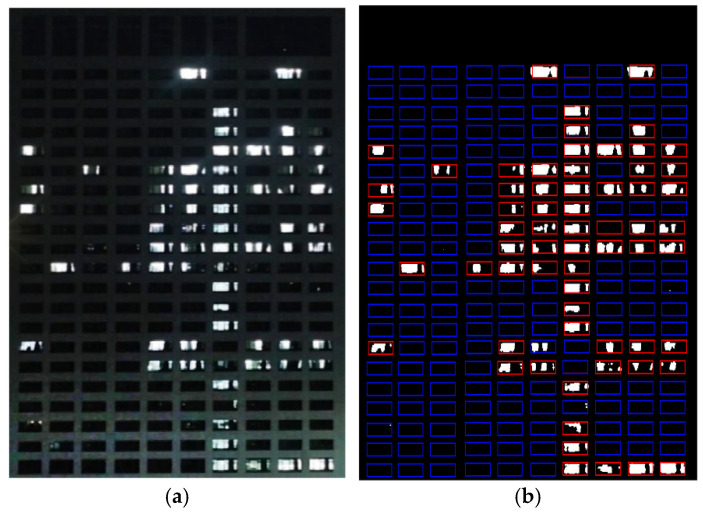
The laboratory usage detected by the proposed algorithm. (**a**) shows the effect of the image obtained by the surveillance camera at night after grayscale processing. The rectangular box in (**b**) shows the positioning coordinates of a laboratory window; the red box indicates that the laboratory is in use, and the blue box indicates that the laboratory is not in use.

The laboratory usage detected by the nighttime detection algorithm at a specific moment is displayed in Figure 14. The rooms with lights on in Figure 14a can be effectively highlighted by red boxes in Figure 14b, while the remaining laboratories, indicated by blue boxes, are not in use. Any changes in the laboratory usage detected by the algorithm are updated and stored in the database for result visualization.

### 3.2. Personnel Detection Based on Indoor Monitoring

**Figure 15:**
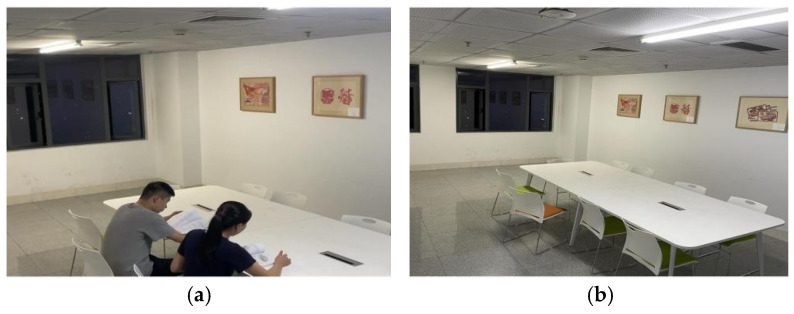

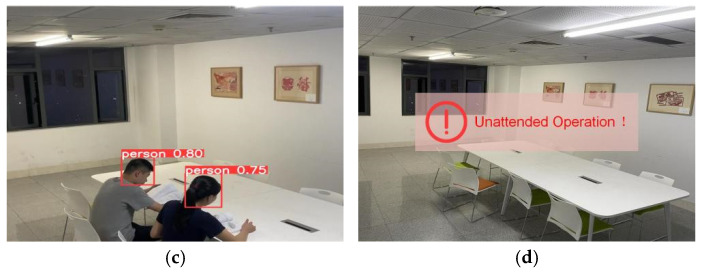
People detection in the room using the YOLO model. (**a**,**b**) show the internal monitoring video of the room in use, (**c**) shows the personnel detection result, indicating their presence, and (**d**) shows the personnel detection result, indicating no people and triggering a warning.

The laboratory personnel detection results of the YOLO model are presented in Figure 15. The people in Figure 15a can be detected and labeled by red marks in Figure 15c. When the laboratory is empty but with lights on (Figure 15b), the system will send a red warning with the words “Unattended Operation”, as presented in Figure 15d.

We record six nighttime test videos under clear weather conditions using a surveillance camera and manually detect the personnel in the laboratory. The manual observations are compared with the algorithm detection results to assess the accuracy of the algorithm. The experimental results are depicted in Table 4.

**Figure 16:**
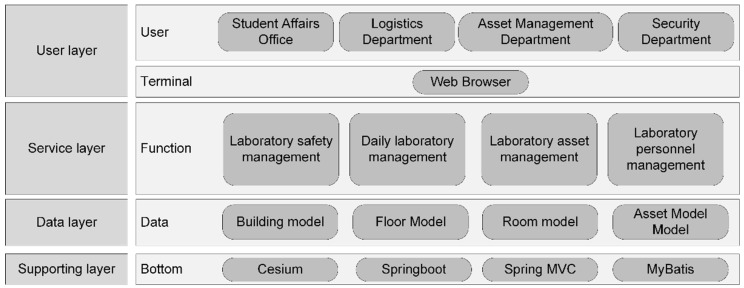
Visualization system architecture of the nighttime usage status detection algorithm.

Table 4 shows that the proposed algorithm achieves an average accuracy of 98.67%, effectively identifying the nighttime usage status of the building room. Additionally, the indoor person detection model precision set reached 94.1%, enabling precise detection of individuals in the room. The visualization system architecture, shown in Figure 16, follows a B/S structure using the SSM (Spring + SpringMVC + MyBatis) framework for database connection.

### 3.3. Multi-Scale 3D Model and Visualization System

**Figure 17:**
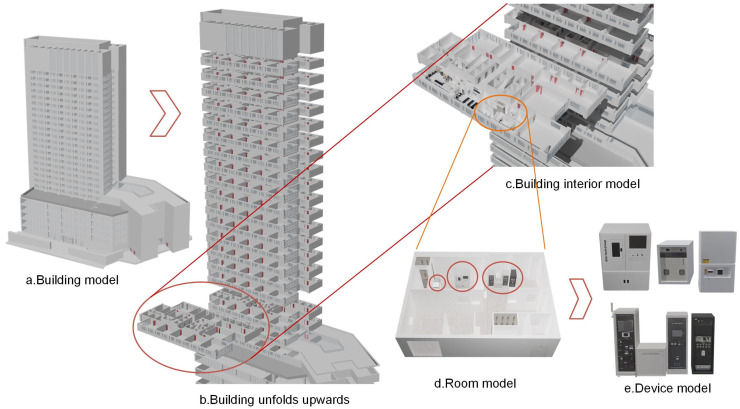
The “Building–Floor–Room” model encompasses the high-rise building model, 3D representations of each floor, and detailed spatial distributions and equipment layouts within the laboratories.

**Figure 18:**
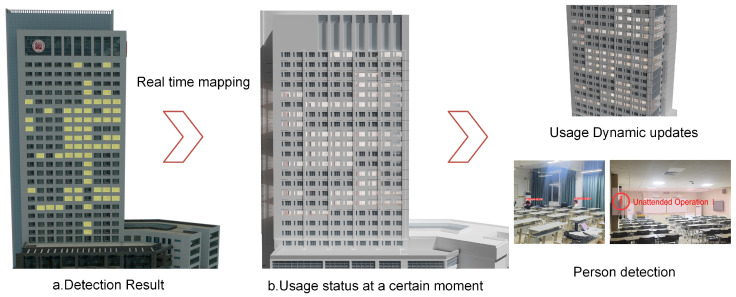
Visualization implementation process for nighttime monitoring in the laboratory.

**Figure 19:**
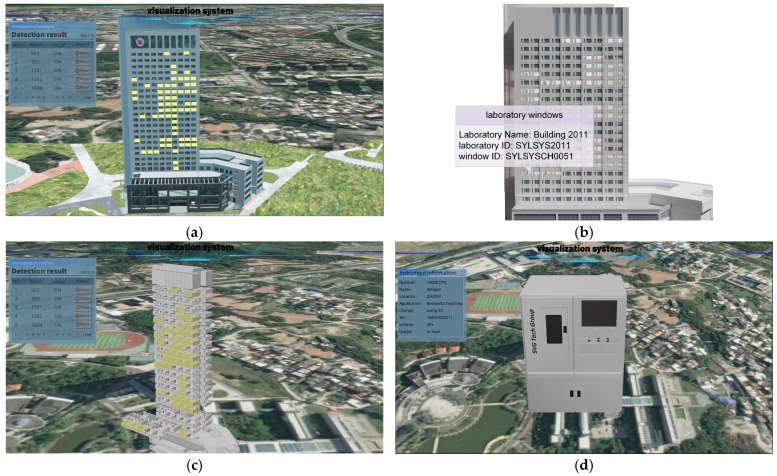
Visualization system interface. (**a**) The nighttime detection result map at a certain moment, (**b**) the indoor asset visualization interface, (**c**) the floor expansion view interface map, and (**d**) the laboratory internal asset query page.

The multi-scale building model, with the “Building–Floor–Room” organization, is depicted in Figure 17. Figure 18 depicts the night monitoring geographic visualization system, which shines and dynamically updates the laboratory model based on occupancy changes. The system monitors personnel in active laboratories and issues warnings regarding energy consumption during prolonged vacancy. Figure 19 shows the system interface diagram. At a certain moment, it shows the nighttime usage situation on a 3D model (Figure 19a), and the highlighted window in the scene represents the laboratory being used. The interface diagram for obtaining room information through space vectors can display attributes such as laboratory ID, window ID, and laboratory name by clicking the trigger to pop up a window (Figure 19b). The system provides a drawer-style extraction display interface, which intuitively analyzes its usage through a model (Figure 19c). Figure 19d provides the query and management interface for assets in the room. The above system functions can achieve vertical management of the laboratory, and unify the management of personnel, finance, and material information.

## 4. Discussion

This study aims to strengthen supervision of nighttime usage of high-rise buildings and detect irregular nighttime use of campus buildings. GIS and BIM are used to address the lack of 3D spatiotemporal data in campus management.

The brightness of building lighting can indicate room usage and be applied to nighttime laboratory use monitoring. Firstly, the brightness is extracted from the outdoor monitoring images of the building and is used for threshold segmentation. The results are matched with the building window templates to determine room occupancy. Experiments show that the algorithm achieves an accuracy of 98.67% in room detection. Compared with wireless facility-based detection methods, this method reduces costs by minimizing the need for additional sensors. However, unattended situations may arise if users forget to turn off the lights. Relying solely on outdoor lighting detection can lead to misjudgment, so YOLOv8 is used to detect human presence, with a trained model precision of 94.1%, enhancing detection reliability. This can help quickly identify and respond to unattended idle energy consumption. System prompts can reduce the frequency of manual inspections and improve management efficiency. However, due to the high mobility of personnel, static people detection may lead to misjudgment.

Relying solely on charts for test result analysis is impractical, as a single display cannot showcase the usage of numerous vertically structured rooms. We reconstructed a 3D model of the campus building based on oblique photography technology and mapped the detection results onto it. In addition, finer-scale BIM models and laboratory instrument models were constructed based on indoor point clouds, enabling full-space visualization of both indoor and outdoor environments. The use of 3D models for visualization in this study presents spatial usage and the dynamic changes in usage. The system can be used for building management.

## 5. Conclusions

Geographic visualization can optimize campus infrastructure and help build a smart campus. Nighttime laboratory usage monitoring is a key means of ensuring campus safety. In this study, we created laboratory building realistic 3D models with different particle sizes and used a “Building–Floor–Room” database to achieve hierarchical and detailed management of a laboratory. First, we reconstructed the 3D models of campus buildings based on oblique photography technology and constructed a multi-scale building model based on indoor point clouds. Then, laboratory usage was extracted from unstructured images through techniques such as template matching, nearest-neighbor clustering analysis, threshold segmentation, and deep learning. Finally, based on the SMM framework, a monitoring system for nighttime laboratory usage was designed. The CZML format was utilized for online data flow, enabling dynamic 3D geographic visualization of the spatiotemporal distribution of laboratory usage at different times. The results indicate that the image processing technology allows for large-scale, cost-effective nighttime supervision of buildings. The application of 3D visualization technology improves campus management by addressing the current gap in 3D spatiotemporal data, which helps with nighttime laboratory monitoring. Spatial visualization can provide strong support for management decisions and help improve campus management efficiency and services.

As the demand for smart campus building management grows, the system’s function needs to be further enhanced. Future research is expected to focus on the following: (1) detecting key information such as changes in personnel numbers and activity frequency, capturing dynamic behavior, and providing more accurate and comprehensive occupancy data; (2) integrating an intelligent control system to detect and evaluate idle energy consumption, regulating energy use; and (3) expanding beyond static facility management to include dynamic equipment tracking and management, addressing the ongoing movement of laboratory equipment.

## Figures and Tables

**Table 1:** Video parameters (from DAHVISION Company).

Parameter	Parameter Values
Resolution	2560 × 1440
Video compression standards	H.265, H.264
Focal length	4.7 mm–94 mm

**Table 2:** NCC template-matching results at different thresholds.

Threshold	Number of Windows	NCC Template-Matching Results	Number of Successful Matches	Matching Accuracy
0.95	190	82	54	28.4%
0.9	190	454	133	70%
0.85	190	1169	170	89.5%
0.8	190	2224	188	98.9%
0.75	190	3621	190	100%
0.7	190	5494	190	100%

**Table 3:** The training parameters of the YOLO model.

Parameter	Experiment Configuration
Operating System	Windows 10 Pro
CPU	Intel(R) Core(TM) i7-8700 (Intel: Santa Clara, CA, USA)
GPU	NIVIDIA GeForce GTX1080Ti (NIVIDIA: Santa Clara, CA, USA)
Deep learning framework	Pytorch 1.8.1

**Table 4:** Comparison of the manual observation and algorithm results.

Number	Duration (Seconds)	Visual Inspection Change Frequency	Algorithm Detection Change Count	Detection Accuracy	Algorithm Validation Results
1	19:10	150	1	1	100%
2	20:10	250	4	4	100%
3	21:10	300	2	2	100%
4	22:10	240	3	3	100%
5	23:10	750	14	13	92.86%
6	00:10	700	15	15	100%

## Data Availability

All data and materials are available upon request.
